# The Causation of Tabes and of General Paralysis

**Published:** 1916-12-09

**Authors:** 


### THE CAUSATION OF TABES AND OF GENERAL PARALYSIS.

### A New Observation from Fiji.

One of the district medical officers of Fiji, Mr. Philip Harper, M.R.C.S., L.R.C.P., has communicated to the *Lancet* * certain observations on the occurrence of tabes dorsalis (locomotor ataxia) and general paralysis of the insane as sequelæ of the tropical disease frambœsia (or "yaws") which seem to have escaped all previous notice, and may turn out to be of great importance. These two nervous diseases, locomotor ataxia and general paralysis, are exceedingly common in this country, and account for much too high a death-rate in our bills of mortality. They are, besides, of a most distressing and disabling nature, and inflict on their victims years—often many years—of invalidism and unproductivity before death seals the fate of the patients. These two diseases have long been known to affect pre-eminently those who have in earlier years suffered from syphilis. About fifteen years ago the opinion was put forward on clinical grounds that the relation is even closer than this; that without pre-existing syphilis there can be neither locomotor ataxia nor general paralysis. This view was later on very strongly confirmed by pathological experts, especially by Mott, and is now generally accepted as a truth established almost beyond the possibility of error. At the same time it has long been known that neither the older anti-syphilitic drugs, mercury and the iodides, nor the newer remedies, salvarsan and the like, have any beneficial influence on the two diseases when once they are established. This clinical finding was taken to mean that though the organism of syphilis is a necessary antecedent cause, it is not the direct exciting factor in locomotor ataxia and general paralysis; and this view was expressed in the adjective "parasyphilitic" applied to the two nervous diseases in question.

### The Causation of Locomotor Ataxia.

The new observations already referred to are to the effect that both tabes and general paralysis may follow the tropical disease frambœsia in patients who have never had syphilis. The peculiar significance of this fact—if fact it be—is that frambœsia is a disease which in some respects, though not in all, bears distinct resemblances to syphilis. It is believed to be caused by a spirochæte, *treponema pertenue*, which is so like that of syphilis that only the most expert bacteriologists can distinguish one from the other. Clinically there are further resemblances, including the Wassermann reaction, and not many years ago the hypothesis was held by many tropical experts that frambœsia was merely syphilis modified by race and climate; Sir Jonathan Hutchinson, for example, long adhered to it. All the most recent authorities, however, on tropical diseases are opposed to this view of the identity of the two infections; and certainly there is a long list of dissimilarities between them.†

* October 14, 1916, p. 678. † See Sir P. Manson's *Tropical Diseases*, Fifth Edition, p. 653.

It does not seem to have struck anyone hitherto to inquire whether the "parasyphilitic" nerve diseases, locomotor ataxia and general paralysis, are also found as sequelæ of frambœsia; obviously such a discovery as that now reported by Mr. Harper is of the highest importance, if it is confirmed and established, and may lead to a reconsideration of the relation between syphilis and frambœsia.

Before it would be right to start speculating on this matter there are some obvious grounds for caution. For one thing, it is conceivable that the six frambœsia patients in whom Mr. Harper found general paralysis and tabes dorsalis may have been at some previous date infected with syphilis as well as with frambœsia; and the nerve lesions may have been due to the former, not the latter. The answer to this objection is, no doubt, that syphilis is unknown amongst the Fijians—an argument which Manson and others have long used against the possible identity of the two diseases. This particular argument as to the non-occurrence of syphilis in Fiji is not convincing when it is brought forward as tending to prove that frambœsia and syphilis are different diseases; for the very hypothesis that they may be the same presupposes that syphilis in Fiji (and the other tropical countries where yaws exists) is a modified form of the disease as known elsewhere.

### Wanted, More Facts.

Be that as it may, it is clear that the identification of tabes and general paralysis as possible sequelæ of frambœsia with no antecedent syphilis must, if it is proved, influence current views about the two infections. It would be of great importance to ascertain whether there is any notable difference between tabes, for instance, following frambœsia and the same disease when it follows syphilis. The case notes which Mr. Harper gives seem to be those of unmistakable advanced tabes and general paralysis, of distinctly severe and rapidly progressive types. This point needs further investigation, as do indeed the whole of the circumstances of these interesting Fijian cases. Incidentally, it may be added that Mr. Harper mentions another suggestive observation—namely, that aneurysm is common in Fiji, although syphilis is believed to be non-existent. Possibly, therefore, frambœsia, which is almost universal among the natives, may predispose to aneurysm, another point of resemblance to syphilis which does not seem to have been noted before. Why no one has ever previously thought of investigating the incidence of aneurysm and of parasyphilitic diseases in communities where syphilis is rare or absent but frambœsia is common is difficult to say. It seems so simple now that Mr. Harper has thought of it, but that must not blind us to the fact that he has made original observations of a nature which may profoundly affect the medical teaching of the day.

